# Impact of Saliva and Cariogenic Microbiota on the Chemotherapy-Induced Oral Mucositis in Oncopediatric Patients: A Preliminary Longitudinal Study

**DOI:** 10.1155/2020/1243953

**Published:** 2020-07-20

**Authors:** Raphael Cavalcante Costa, Paula Maria Maracajá Bezerra, Lecidamia Cristina Leite Damascena, Isabella Lima Arrais Ribeiro, Paulo Rogério Ferreti Bonan, Simone Alves de Sousa, Leopoldina de Fátima Dantas Almeida, Ana Maria Gondim Valença

**Affiliations:** ^1^Department of Prosthodontics and Periodontology, Piracicaba Dental School, University of Campinas (UNICAMP), Piracicaba, São Paulo, Brazil; ^2^Department of Clinical and Social Dentistry, University of Paraíba (UFPB), João Pessoa, Paraíba, Brazil; ^3^Department of Statistics, Federal University of Paraíba (UFPB), João Pessoa, Paraíba, Brazil; ^4^Department of Social Medicine, School of Medicine, University of São Paulo, Ribeirão Preto, Brazil

## Abstract

This study aims to evaluate the salivary parameters and cariogenic microbiota of pediatric oncological patients and their correlation with severe oral mucositis (SOM). A preliminary longitudinal study included patients in the age range from 4 to 18 years (*n* = 26), with diagnosis of primary cancer, who were followed up before and after time intervals of two, five, and ten weeks of induction chemotherapy. Oral mucosa examinations were performed by means of the modified Oral Assessment Guide (OAG) by calibrated examiners (*κ* > 0.70). Saliva analysis (unstimulated saliva flow (USF), clinical saliva viscosity (CSV), and pH) and microbiological (total *Streptococcus* (TS) and *Streptococcus* of the *mutans* group (SMG)) tests were performed using unstimulated saliva. The data were analyzed by the Wilcoxon and Spearman Correlation tests (*α* = 5%). The patients were predominantly of the female sex (*n* = 15; 57.7%), adolescents (*n* = 15; 57.7%), and patients with hematological tumors (*n* = 21; 80.8%). SOM was more frequent in the tenth week (*n* = 7; 28.6%). The values of USF, CSV, pH, TS, and SMG were not changed by the institution of chemotherapy (*p* > 0.05). These values were correlated with SOM and the time, TS and SMG, and CSV and SMG. The salivary and microbiological parameters investigated did not influence the severity of oral mucositis in the pediatric patients oncological..

## 1. Introduction

Childhood cancer is a chronic and degenerative disease that is considered a serious and relevant public health problem [1]. Annually, about 20 million childhood cancers are diagnosed worldwide [2] with a projected 30% increase of cases to be identified and treated up to 2020 [3]. Although childhood cancer treatments are improving, this disease is still the main cause of mortality in children and adolescents in the age group from 1 to 19 years and has a negative impact on the health services economy [4].

Antineoplastic therapy exposes the patients to numerous comorbidities, making the cancer treatment longer and debilitating with additional costs and reducing the quality of life [2]. Oral tissues are profoundly affected by the toxicity of antineoplastic agents producing numerous comorbidities such as mucositis, reduced salivary flow, and opportunistic viral and fungal infections [5]. Among these comorbidities, several studies [5–9] have considered oral mucositis (OM) as the main complication of the clinical condition of pediatric oncological patients. OM lesions are characterized by inflammatory, ulcerative, and bleeding areas with painful clinic symptoms that are difficult for treatment with topic or systemic medications [10]. Commonly, OM manifests clinically 3 to 10 days following the start of chemotherapy; it depends on the kind of cancer and treatment procedure [11].

Oncopediatric patients are more vulnerable to oral mucositis than adult patients because of the immaturity of tissues and organs, and the immune system is still in the process of formation [6]. As OM cases are more severe in children, they compromise more rapidly speech, swallowing, oral hygiene and, consequently, delay treatment and increase mortality [12]. Approximately 80% of children and adolescents manifest OM at some stage of treatment [11]. For this reason, knowing the factors that may be related to the occurrence, progression, and duration of OM will aid in the development of more effective preventive and therapeutic protocols [8].

Currently, the pathogenesis of oral mucositis is still not well understood [10]. It is known that saliva and oral biofilm are risk factors for inflammation and ulceration of the lesions [10, 13–15]. Saliva facilitates the adhesion of the microorganisms as well as it is the energy source for the development of the biofilm. The bacteria present in this biofilm produce metabolic byproducts that stimulate the production of proinflammatory cytokines (IL-1*β*, IL-6, and TNF-*α*). These factors make tissue repair more difficult, increasing remission time, risk of systemic infection, and healing of OM [10].

In pediatric patients, these changes may be exacerbated since the synthesis and composition of saliva are physiologically modified during growth (e.g., salivary gland development, bone synthesis and maturation, exfoliation, and dental eruption) [16]. In addition, there is evidence that children on chemotherapy have a higher incidence of dental caries due to changes in the colonization of specific bacterial groups during treatment [16–18]. Several studies have been carried out with cancer patients on radiotherapy, but studies evaluating the impact of chemotherapy, the main treatment of childhood cancer, on the quantitative and qualitative aspects of saliva, and cariogenic biofilm and its possible relation with the aggravation of oral mucositis in children and adolescents are limited.

Therefore, the aim of this study was to investigate whether saliva and cariogenic microbiota would be related to the greater severity of OM in children and adolescents submitted to chemotherapy. Our hypothesis is that there exist critical periods of chemotherapy treatment with a greater impact on the oral cavity and, consequently, these modifications (salivary and microbiological) can increase the severity of OM.

## 2. Methodology

### 2.1. Study Design

This preliminary longitudinal study (prospective, clinical, and laboratory) was approved by the Ethics Committee at Federal University of Paraíba (CAAE number: 45800415.7.0000.5188). Patients with a diagnosis of primary cancer were recruited between February 2016 and April 2017 at the Pediatric Oncology Department of Napoleão Laureano Hospital (João Pessoa, Brazil). Detailed information about the study was provided to all participants and responsible adults before obtaining a written consent.

### 2.2. Participant Selection

**Figure 1:**
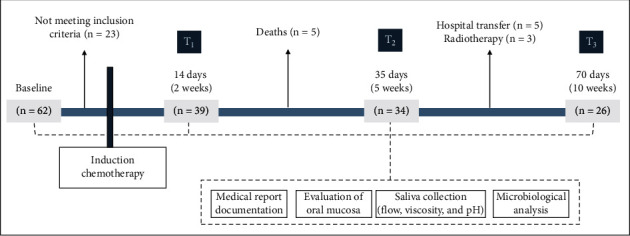
Flow chart of the study.

In view of the low incidence of childhood cancer [4], nonprobabilistic and convenience sampling was adopted. Patients aged 4–18 years were selected according to the inclusion/exclusion criteria (Table 1) and were evaluated longitudinally before and after the diagnosis of cancer (70 days). Thus, a total of 26 patients of both sexes with malignant tumors treated only with chemotherapeutic protocols were included in the final sample. The experimental design of this study can be seen in Figure 1.

### 2.3. Evaluation of Outcome Variables

#### 2.3.1. Medical Report Documentation

The sociodemographic aspects sex, age, skin color (self-reported), and the clinical aspects about the tumor type (hematological or solid) and antineoplastic treatment instituted (chemotherapeutic class) were obtained from the medical record charts of the above mentioned hospital. The chemotherapy treatments used were of the (a) miscellaneous class, (b) natural products, (c) antimetabolites, and (d) aquilant agents (Classification of the National Sanitary Vigilance Agency/Agência Nacional de Vigilância Sanitária (ANVISA), Brazil), in accordance with the therapeutic protocols of the hospital described in our previous studies [6, 8].

#### 2.3.2. Clinical Evaluation of the Oral Mucosa

**Figure 2:**
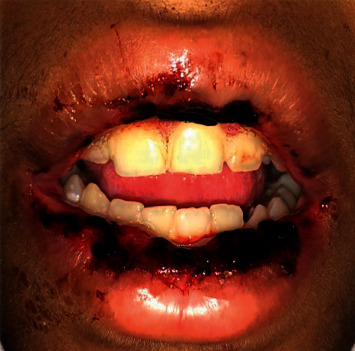
Example of severe oral mucositis (SOM) in the patients of this study (score 3-labial mucosa).

Oral mucositis was diagnosed and classified by the modified OAG [13]. This index evaluates compromised oral sites and functions by attributing values from 1 to 3 according to the severity of the inflammatory tissue reaction. The exams for diagnosing mucositis were performed in the dental office and/or hospital beds, with the use of mouth mirrors and headlamps as a source of illumination, by two (RCC and TVC) previously calibrated examiners (*κ*  > 0.70). In this study, the dependent variable “oral mucositis” was grouped and dichotomized into the nominal categories without oral mucositis or light oral mucositis (scores 1 and 2—OAG) and severe oral mucositis (SOM) (score 3—OAG). SOM was indicated when some of the 8 features (voice, swallowing, lips, tongue, saliva, oral/palate mucosa, labial mucosa, and gingiva) of which the OAG is composed was identified with score value “3” (Figure 2).

#### 2.3.3. Saliva Collection

Saliva collections occurred before baseline and after 2 (T1), 5 (T2), and 10 (T3) weeks of induction chemotherapy. These time intervals were established based on previous studies that identified them as critical periods for the onset of severe oral mucositis [6, 8]. Unstimulated saliva was obtained by the method of actively spitting into a previously sterilized receptacle. This methodology is recommended for verifying cases of hyposalivation because it is subject to a lower influence of external factors [14, 18, 19]. Due to operational questions, and seeking to minimize the influence of the circadian rhythm of salivation, collections were always made in the morning period, 1 hour after the last time of tooth brushing and eating, under the same conditions of positioning, lighting, and sounds [18, 19]. Initially, the patients were asked to remain seated, in a 90° position, without speaking and moving, with the upper limbs resting on the lower limbs. After swallowing all the saliva in the mouth, the head was lowered to 45°, with eyes focused on a fixed point. In a silent environment, saliva was collected for a period of 2 minutes.

After collection, saliva was used to determine (a) nonstimulated salivary flow, (b) clinical salivary viscosity, and (c) salivary pH. The salivary flow was measured in mL of saliva produced every 1 minute (mL/min). The clinical viscosity of saliva was determined by measuring (mm) the thread of saliva formed from aliquots of 200 *μ*L submitted to vertical traction by means of glass slides. The assay was performed in triplicate, and the mean value obtained in each sample was determined [19, 20]. Afterward, the saliva pH was evaluated with a digital pH meter (Voltcraft, Berlin, Germany) previously calibrated.

#### 2.3.4. Cariogenic Microbiota Analysis

Cariogenic bacteria present in the saliva were evaluated in selective culture media. For the analysis of total *Streptococcus* (TS), the traditional technique of cultivation on Mitis Salivarius Agar (MSA) (Difco, São Paulo, Brazil) was used. *Streptococcus* of the *mutans* group (SMG) was evaluated with the MSA supplemented with 20% of sucrose, 1% potassium tellurite, and 0.2 UI of bacitracin (MSB) [20, 21]. Initially, aliquots of 20 *µ*L of saliva were inserted into 180 *µ*L of sterile NaCl solution (0.9%) and then homogenized in a solution agitator (Phoenix, São Paulo, Brazil). Serial dilutions of the samples were made immediately. The drop technique [21, 22] was adopted for seeding the plates. Posteriorly, the plates were incubated in a bacteriological oven (±37°C/48 h). As a negative control, 0.9% NaCl solution was cultivated in the culture media previously described. Finally, the microorganism counts per milliliter of saline solution (CFU/mL) were performed by a single previously trained researcher (RCC). The catalase test and Gram staining were performed to confirm the bacterial growth of the *Streptococcus*.

### 2.4. Statistical Analysis

The data were submitted to descriptive and inferential analysis using the statistical program IBM-SPSS®, version 20.0 (License 1989.2011). The numerical variables were initially evaluated with regard to their normality and homoscedasticity by the Shapiro–Wilk and Levene tests (*α* = 5%). In view of the data distribution, oral mucositis and the salivary (flow, viscosity, and pH) and microbiological variables (TS and SMG) were analyzed by the Wilcoxon test. For this analysis, the medians of the variables tested in each time interval of evaluation were compared, considering the sample as being unpaired due to the losses that occurred during the follow-up. The Spearman correlation was used to establish correlations between the presence of SOM and the salivary and microbiological parameters. The level of significance adopted for all analyses was 0.05% for type I error. The statistical power was calculated using the software G-Power version 3.1.9.2 considering the main variables of the study (β> 0.8, α= 0.05).

## 3. Results

### 3.1. Sample Characterization

In this study, the sample was comprised of 26 oncopediatric patients, predominantly of the female sex (*n* = 15; 57.7%), adolescent (*n* = 15; 57.7%), and white skin color (*n* = 11; 42.3%). The mean age was 11.1 (±4.8) years. With regard to diagnosis, hematological tumors were the type most frequently identified (*n* = 21; 80.8%), with higher prevalence of acute lymphoblastic leukemia (*n* = 11; 42.3%), followed by acute myeloid leukemia (*n* = 4; 15.4%), non-Hodgkin's lymphoma (*n* = 4; 15.4%), and osteosarcoma (*n* = 4; 15.4%). During follow-up, the agents most frequently administered in the 2nd week (58.8%) and 5th week (44.7%) were of the class of natural products, and in the 10th week, the antimetabolite agents (40.0%).

### 3.2. Severe Oral Mucositis (SOM)

**Figure 3:**
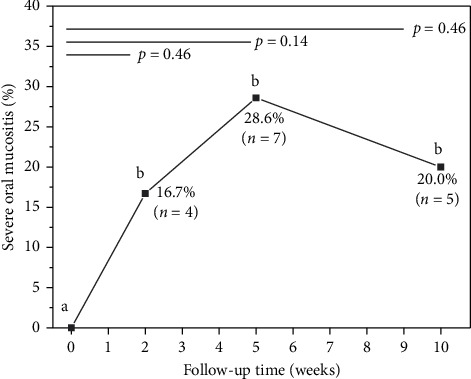
Graphical distribution of the median of patients diagnosed with SOM at each follow-up time. Different letters indicate statistical difference (Wilcoxon test, *p* < 0.05).

SOM cases occurred in all evaluated weeks: 2nd (*n* = 4; 16.7%), 5th (*n* = 7; 28.6%), and 10th (*n* = 5; 20.0%) (Figure 3). In these periods, the jugal mucosa and palate mucosa were the sites most affected by SOM. There was a trend towards an increase in the number of cases of SOM in the 5th week; however, these differences were not shown to be significant between the time intervals evaluated after beginning with chemotherapy (*p* > 0.05).

### 3.3. Salivary Outcomes

**Figure 4:**
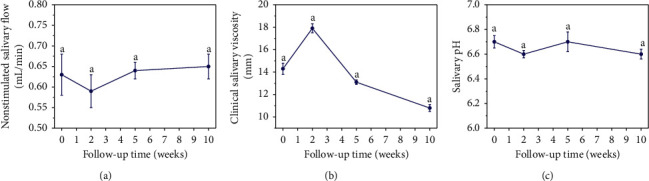
Graphical distribution of the median (standard deviation). (a) Nonstimulated salivary flow (mL/min), (b) clinical salivary viscosity (mm), and (c) salivary pH during follow-up periods. No statistically significant differences were found (Wilcoxon test; *p* > 0.05).

Figure 4 shows the distribution of unstimulated salivary flow (USF-Figure 4(a)), clinical saliva viscosity (CSV-Figure 4(b)), and salivary pH (Figure 4(c)) before and during the 2nd, 5th, and 10th week of chemotherapy treatment. A trend towards reduction in USF was observed in the 2nd week (0.59 mL/min). Afterwards, there was a progressive increase in the 5th (0.64 mL/min) and 10th week (0.65 mL/min); however, no statistically significant differences were found before and during treatment (*p* > 0.05). CSV was another saliva parameter evaluated, showing no significant differences (*p* > 0.05) between the time intervals of evaluation. The mean value before treatment was 14.3 mm, with a trend towards saliva becoming more viscous in the 2nd week (17.9 mm), decreasing in the 5th week (13.1 mm), and becoming more fluid in the 10th week (10.8 mm). In addition, the chemotherapy had no influence on the pH of saliva , which remained close to neutrality (*p* > 0.05).

### 3.4. Cariogenic Microbiota Outcomes

**Figure 5:**
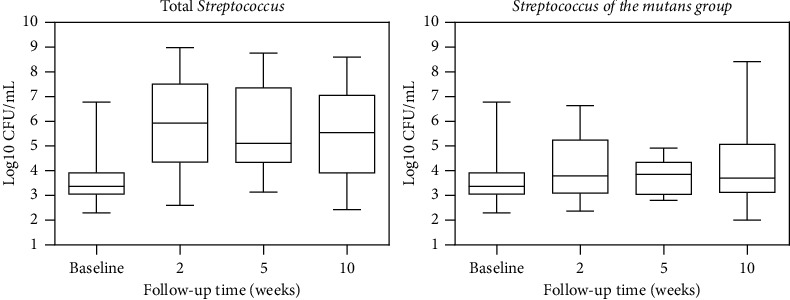
Graphical distribution of mean cell viability (CFU/mL) of total *Streptococcus* (TS) and *Streptococcus* of the *mutans* group (SMG) during follow-up periods. No statistically significant differences were found (Wilcoxon test; *p* > 0.05).

The microbiological analysis of the saliva of child and adolescent oncology patients is shown in Figure 5. The amount of TS found in the saliva was higher than that of SGM, increasing progressively over the period evaluated. However, no significant differences were found for the cell viability of TS and SMG before and after chemotherapy was instituted (*p* > 0.05).

### 3.5. Correlation

The correlation between the SOM and independent variables (salivary and microbiological outcomes) of this study is described in Table 2. The occurrence of SOM was found to be positively correlated only with the time of treatment and was considered a low correlation (*r* = 0.242).

## 4. Discussion

This study brings new insights into the pathophysiology of chemo-induced oral mucositis in oncopediatric patients. We have shown that physicochemical factors of saliva and the cariogenic microbiota are hardly influenced by systemic chemotherapy. Consequently, the impact on the progression of oral mucositis lesions is minimal, and these lesions develop throughout the treatment without critical periods of greater occurrence. Thus, our initial hypothesis that these variables (salivary and cariogenic microbiological) would be altered by chemotherapy and would increase the progression of oral mucositis was rejected.

From this perspective, it was observed that MOG had been diagnosed in all the follow-ups, with incidence varying from 16.7% to 28.6%, as reported in other studies [7, 23–27]. In contrast, higher incidence ranging from 35.5% to 46.0% are also reported [5, 27–30]. These differences may be related to classification by different indices;, the heterogeneity of the sample related to sex and age;, and the periods of treatment investigated, since most of the studies were cross-sectional and retrospective. Additionally, the cumulative effects of chemotherapy on oral tissues may induce clinical manifestations of mucositis in periods of nonspecific latency and immunosuppression [29, 30].

Another relevant point is that few studies describing the location of mucositis lesions are available in the literature. In this study, the jugal mucosa and palatal mucosa were the most frequently affected areas of the oral cavity, corroborating with previous studies [7, 13]. The histology of the mucosa of these regions presents a thin epithelial layer that has been considered intraoral areas more difficult to be hygienic and professional monitored, and this could have potentiated the host cellular damages and increased the severity of mucositis. These facts reinforce the need for constant dental care during chemotherapy treatment, especially in pediatric patients. The children do not have the same attention to oral care than adults, which made early diagnosis difficult and, consequently, increased the progression of the lesions [8].

With respect to the salivary parameters, some studies reported that chemotherapy reduces salivary flow [14, 28–31]. However, the results found in this study diverged from this pattern and corroborate with another study [16]. Firstly, this difference can be attributed to the innumerable therapeutic protocols evaluated, which have different mechanisms of action [5]. Overall, the impact of chemotherapeutic agents on salivary physiology is still not fully understood, making it difficult to compare studies with different protocols. Secondly, psychological aspects such as stress and anxiety are also associated with hyposalivation and may influence the test results [32]. Considering the clinical salivary viscosity, this was not shown to be variable over the course of antineoplastic therapy, which is similar to the results found in children with leukemia during treatment [33].

Some studies indicate the tendency of saliva to become more acidic in the oral cavity after the start of chemotherapy [31, 34]. Here, the salivary pH remained around 6.7, even during chemotherapy. Based on these results, we speculated that a possible reduction in pH could be attributed to unsatisfactory oral conditions and not be directly related to chemotherapies. For instance, episodes of vomiting are frequent during oncologic therapy, and when this secretion remains in the mouth, it becomes the main factor responsible for environmental acidification [1]. Saliva should behave similarly to other body fluids such as blood and urine that maintain the pH stable even under long chemotherapeutic regimens [33–35].

In our experiment, the bacterial colonization of *Streptococcus* did not change after induction chemotherapy. It has been shown that pediatric patients had better oral health than healthy children [36, 35] and rural residents [37] with the same age due to the greater dental care provided during the antineoplastic treatment. The professional care associated with regular oral hygiene interferes with microbial colonization, reducing cariogenic bacterial counts [37, 38]. Moreover, because the *Streptococcus* have aciduric and acidogenic properties [20, 21], they may be less susceptible to the changes in the oral cavity resulting from chemotherapies toxicity. This is a positive topic since a large reduction of oral bacteria could provide greater fungal colonization. This fact may be a possible explanation for the low prevalence of fungal infections such as candidiasis in pediatric patients when compared to adult patients [5].

The correlation analysis showed that there was no relation between the salivary and microbiological parameters evaluated with oral mucositis severity. This result was expected because the chemotherapy institution did not have an impact on saliva quantification (flow, pH, and viscosity) and *Streptococcus* colonization. In addition, only the physicochemical and microbiological characteristics of saliva were analyzed, and the biochemical, hormonal, and immunological aspects, as well as other oral bacteria and fungi that could also influence the severity of oral mucositis were not evaluated. Hence, new studies are encouraged to evaluate the impact of other oral microbial on the mucositis pathogenesis in pediatric patients.

This study presents some limitations, such as the reduced size of the sample that should be taken into consideration when interpreting the above findings. Age, neoplasms, and types of different treatments should also be considered. In addition, there are limitations regarding the biochemical parameters tested (i.e., salivary cytokine or antioxidant levels were not evaluated) and the bacteria count (bacteria detection apparatus was not used). Nonetheless, it is important to emphasize that this report is a preliminary longitudinal follow-up, which evaluated for the first-time oncopediatric patients, considering only severe cases of oral mucositis induced by chemotherapy for long-time (70 days), evidencing their correlation with salivary parameters (flow, viscosity, and pH) and cariogenic bacteria (total bacteria and *Streptococcus*) not previously associated in other studies. Altogether, our preliminary results may serve as a guide for further clinical studies in pediatric oncology field.

In conclusion, the preliminary report showed that salivary and microbiological parameters analyzed before the chemotherapy institution were not changed in the first 10 weeks of treatment and, consequently, not related to the increase in the severity of oral mucositis in pediatric oncological patients.

## Figures and Tables

**Table 1:** Inclusion and exclusion criteria.

Inclusion criteria:
General
Age between 4 and 18 years
Diagnosis of primary malignant neoplasm
No uncontrolled motor and psychological disturbances
Not having started chemotherapy
Only present chemotherapy only as a treatment in the first month
Perform all treatments in the Napoleão Laureano Hospital
Local
Healthy oral mucosa
Ability to spit saliva
Exclusion criteria
Lesions of the oral mucosa before the chemotherapy
Local irradiation history (head and neck)
Inadequate oral hygiene
Children who can not spit saliva
Termination or transfer of treatment to another hospital within 70 days
Terminal critical patients
Presence of xerostomia (baseline)
Patients with restarted treatment for recurrent neoplasia
Non-collaborative patients

**Table 2:** Correlation between study variables.

		SOM	Time	Flow	Viscosity	pH
SOM	Correlation coefficient (*r*)	1,000	—	—	—	—
	*p* value	—	—	—	—	—
Time	Correlation coefficient (*r*)	**0.242 ** ^A^	1,000	—	—	—
	*p* value	**0.021**	—	—	—	—
Flow	Correlation coefficient (*r*)	0.045	0.022	1,000	—	—
	*p* value	0.676	0.837	—	—	—
Viscosity	Correlation coefficient (*r*)	0.018	−0.044	−0.136	1,000	—
	*p* value	0.864	0.678	0.197	—	—
pH	Correlation coefficient (*r*)	−0.194	−0.157	−0.121	0.155	1,000
	*p* value	0.070	0.138	0.257	0.144	—

^A^Statistically significant correlation (*p* < 0.05).

## Data Availability

The data used to support the findings of this study are included within the article.
